# Overcoming challenges to data quality in the ASPREE clinical trial

**DOI:** 10.1186/s13063-019-3789-2

**Published:** 2019-12-09

**Authors:** Jessica E. Lockery, Taya A. Collyer, Christopher M. Reid, Michael E. Ernst, David Gilbertson, Nino Hay, Brenda Kirpach, John J. McNeil, Mark R. Nelson, Suzanne G. Orchard, Kunnapoj Pruksawongsin, Raj C. Shah, Rory Wolfe, Robyn L. Woods, on behalf of the ASPREE Investigator Group

**Affiliations:** 1 Department of Epidemiology & Preventive Medicine, Monash University, ASPREE Co-ordinating Centre, 99 Commercial Road, Melbourne, VIC 3004 Australia; 2 School of Public Health, Curtin University, Perth, WA Australia; 3 Department of Pharmacy Practice and Science, College of Pharmacy and Department of Family Medicine, Carver College of Medicine, The University of Iowa, Iowa City, USA; 4 Chronic Disease Research Group, Minneapolis Medical Research Foundation, Minneapolis, Minnesota USA; 5 Berman Center for Outcomes and Clinical Research, Hennepin Healthcare Research Institute (HHRI), Hennepin Healthcare, Minneapolis, MN USA; 6 Menzies Institute for Medical Research, University of Tasmania, Hobart, TAS Australia; 7 Department of Family Medicine and Rush Alzheimer’s Disease Center, Rush University Medical Center, Chicago, IL USA

**Keywords:** Health data, Clinical trial, Data quality, Health technology

## Abstract

**Background:**

Large-scale studies risk generating inaccurate and missing data due to the complexity of data collection. Technology has the potential to improve data quality by providing operational support to data collectors. However, this potential is under-explored in community-based trials. The Aspirin in reducing events in the elderly (ASPREE) trial developed a data suite that was specifically designed to support data collectors: the ASPREE Web Accessible Relational Database (*AWARD*). This paper describes *AWARD* and the impact of system design on data quality.

**Methods:**

*AWARD*’s operational requirements, conceptual design, key challenges and design solutions for data quality are presented. Impact of design features is assessed through comparison of baseline data collected prior to implementation of key functionality (*n* = 1000) with data collected post implementation (*n* = 18,114). Overall data quality is assessed according to data category.

**Results:**

At baseline, implementation of user-driven functionality reduced staff error (from 0.3% to 0.01%), out-of-range data entry (from 0.14% to 0.04%) and protocol deviations (from 0.4% to 0.08%). In the longitudinal data set, which contained more than 39 million data values collected within *AWARD*, 96.6% of data values were entered within specified query range or found to be accurate upon querying. The remaining data were missing (3.4%). Participant non-attendance at scheduled study activity was the most common cause of missing data. Costs associated with cleaning data in ASPREE were lower than expected compared with reports from other trials.

**Conclusions:**

Clinical trials undertake complex operational activity in order to collect data, but technology rarely provides sufficient support. We find the *AWARD* suite provides proof of principle that designing technology to support data collectors can mitigate known causes of poor data quality and produce higher-quality data. Health information technology (IT) products that support the conduct of scheduled activity in addition to traditional data entry will enhance community-based clinical trials. A standardised framework for reporting data quality would aid comparisons across clinical trials.

**Trial registration:**

International Standard Randomized Controlled Trial Number Register, ISRCTN83772183. Registered on 3 March 2005.

## Background

Results from clinical trials often form the backbone of clinical guidelines. Therefore, it is essential that trials produce high quality data by minimising erroneous and missing data [1]. Current strategies for improving data quality focus on trial design, conduct and governance [2, 3], and limiting data collection to essential items in order to restrict the potential for inaccurate and missing data [2, 4–6]. Hospital-based trials can limit research data collection by leveraging existing data within the electronic health record (EHR) [7]. However, community-based trials operate outside the data infrastructure. For complex, large-scale community-based trials, data quality hinges upon data collectors accurately administering questionnaires and conducting assessments with participants, abstracting data from clinical records when relevant and where available, and executing processes to follow up unusual data and event triggers. This is a challenging remit and carries risks for data quality.

Poor data quality arises for a variety of reasons including insufficient operational support for data collections [8], complex data abstraction procedures requiring data collectors to interpret and adhere to definitions [2, 8–11] and manual calculation of values [12]. While modern data collection systems have been shown to improve data quality through improved data validation [13], they are only effective to the extent that they are intuitive and the end-users can readily work with the system. Indeed, the extent to which technology can be used efficiently, effectively and satisfactorily by users has been an important limiting factor in the update of EHR systems [14, 15]. Poor usability has been linked to inefficiency, frustration, confusion and stress for study staff [15, 16], which may negatively impact data quality. Conversely, improvements in usability have been shown to improve data quality and reduce errors in a hospital setting [17, 18]. However, to the knowledge of the authors no in-depth study of the role and impact of technology in quality assurance for trials has been conducted in the community.

The ASPirin in Reducing Events in the Elderly (ASPREE) study was a community-based randomised, double-blinded, placebo-controlled multi-centre trial (*n* = 19,114) of daily 100 mg enteric-coated aspirin in healthy community-dwelling older adults in Australia (*n* = 16,703) and the USA (*n* = 2411). ASPREE included in-person data collection at baseline visits, quarterly phone calls throughout follow up and an average of five annual data collection visits conducted at community venues, general practice clinics and clinical trial centres (~ 1990 data values per participant in total). Data collection included physical and lifestyle measures, personal and family history, measurement of haemoglobin, fasting glucose, creatinine, lipid panel and urinary albumin:creatinine ratio, a cognitive battery, mood and depression questionnaire, physical function measures (gait speed and grip strength), Katz activities of daily living, quality of life and clinical endpoint screening (death, dementia, disability, cancer, cardiovascular disease, depression and major haemorrhage) [19, 20]. All endpoint triggers required supporting clinical documentation (manually retrieved by ASPREE staff from health services) and subsequent adjudication by a panel of clinical experts. This activity occurred across 43 study sites and involved more than 900 office-based and in-field data collectors.

ASPREE required a data collection system that could support a wide range of activity that was in addition to direct data entry and included the precise tracking of staff activity “in the field”, study medication, accuracy of measurement devices, venues for study visits (e.g. primary care practices), vehicles (and their availability for staff activity) and follow up of clinical events. At the time of study commencement there was no “off the shelf” commercial or freeware software meeting these requirements. The data collection system used for the ASPREE pilot study [21] consisted of simple data entry web forms with limited validation. To support ASPREE, this system was expanded to support key operational processes (e.g. participant recruitment, correspondence, visit scheduling, event detection) in addition to data collection. By shaping the system in consultation with, and anticipating the needs of, data collectors, it was hypothesised that the improved operational support would produce higher-quality data. The final result was a sophisticated, flexible, modular data solution called the ASPREE Web Accessible Relational Database (*AWARD*) suite. In this paper we discuss four known challenges to data quality challenges that were identified in the literature and confirmed through consultation with data collectors, and present the design solution for each challenge implemented in *AWARD*. The positive impact of these solutions on data quality is presented through a comprehensive account of the quality of *AWARD* longitudinal data.

## Method

### Developing *AWARD* system requirements

Key operational requirements identified via a needs assessment included study visit venue (medical practice or community venue) room booking, participant visit booking, tracking and conduct of 3-monthly retention calls, tracking of study medication bottles, retention of participants at risk of withdrawal, communication with primary care physicians, staff decision and protocol adherence support, data entry of primary study data, entry of operational data such as contact details, and maintenance of confidentiality by providing different levels of access so that access to identifying information was limited to site staff. A design solution was implemented for each key requirement (see Table 1) by systematically upgrading the ASPREE pilot [21] system that had been developed in house between 2002 and 2003. Completion of major upgrades occurred over 12–15 months. During this time the simple ASPREE pilot web forms were utilised for data collection. Over the next 3 years additional modules for general practitioner recruitment and event adjudication were deployed.

**Table 1:** Operational and data management considerations and solutions

Operational domain	Key requirements	Design solution
Visit booking	- Identification of participants to be booked and the visit required - Organisation of “due” participants by visit venue - Computation of visit venue booking time - Recording and tracking of venue room bookings - Recording tracking of participants bookings linked with room bookings	“2 step” solution implemented - Venue room booking information entered via a single web page - Participants’ booking information entered on a nested web page Bookings presented in both calendar and list format via the web application
Conduct of calls	- Identification of participants to be contacted - Mechanism to record call attempts and messages - Mechanism to record if participants are unavailable for calls at certain timepoints	Online call tracking implemented - List of participants due and eligible to receive calls available via web application Simple online phone call data collection form
Study medication tracking	- Tracking of dispensing and retrieval of study medication bottle - Mechanism to ensure that the correct medication is provided to each participant	Online drug log implemented - Study medication bottle dispensation date and retrieval date recorded - Pill count logged To avoid unnecessary queries caused by transcription errors, each participant’s unique study medication code prompted and validated on data entry
Retention	- Conduct of scheduled contact at certain timepoints identified as increasing the risk of participant withdrawal (e.g. between dementia trigger and completion of additional cognitive assessment) - Mechanism required to shift participants at risk of withdrawal from the regular contact lists to a retention team list	Retention status implemented - Database “views” utilised to derive a status describing whether scheduled study contact was appropriate (e.g. not eligible for phone contact – dementia trigger follow up in progress) Status utilised to shift participants from regular contact lists to retention team lists
Communication	- Mechanism for staff to notify PCPs/GPs of abnormal results - Mechanism for requesting clinical documents from third parties (e.g. hospitals, specialists and general practitioners)	Curated third party communication pipeline created and implemented - Standard document request and abnormal result notification letters auto-populated with relevant participant details via web application - Microsoft Visual Basic for Applications utilised to send standard letters via fax or email communications
Staff decision support	- Mechanism to ensure that protocol specified follow up of endpoints was completed - Mechanism to ensure protocol specified follow up of abnormal results was completed - Mechanism to ensure that only eligible participants were randomised	Key operational “status” for each study participant or key step derived and displayed - Database views utilised to derive a status describing the operational “next step”’ (e.g. event coded – awaiting supporting documents; annual visit – overdue etc) Status displayed on relevant pages on the user interface Randomisation restrictions implemented - Automated checks compared entered data against eligibility criteria - Randomisation function disabled for ineligible participants
Data entry	- Mechanism to alert staff to potentially incorrect data for review - Clear process for alerting staff to data queries for resolution	Checks and balances implemented to minimise transcription errors - Pre-programmed value ranges, process prompts and protocol compliance checks, checked at the point of data entry - Page submission restrictions implemented to check for logic between values on a page Staff action list implemented - Automated checks compared entered data against acceptable ranges^a^ and produced “action items” - Staff specific list of action items displayed on “home” page of *AWARD*-Data web application

*PCP* primary care provider, *GP* general practitioner

^a^Acceptable ranges were determined by an expert committee based on physiological plausibility

### Overview of the *AWARD* suite

**Fig. 1:**
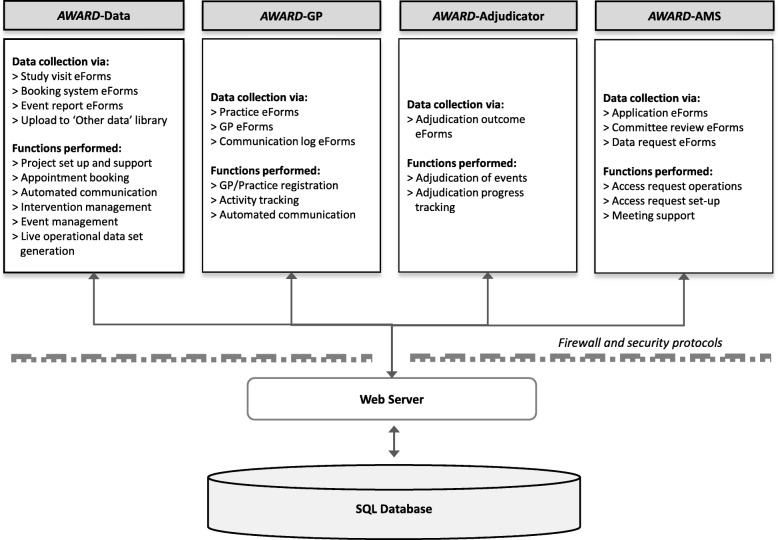
Conceptual design and functionality of the ASPREE Web Accessible Relational Database (*AWARD*) suite. e-forms = electronic versions of case report or other forms. “Other data library” refers to the library storing unstructured files such as PDF supporting documents, PDF consent forms and retinal photographs

The *AWARD* suite consisted of four communicating modules, each with a particular focus and a specific group of users: *AWARD*-Data, *AWARD*-General Practice (*AWARD*-GP), *AWARD*-Adjudicator and *AWARD*-Access Management System (AMS) (Fig. 1). *AWARD*-Data was the first module deployed (in 2010) for on-site and in-field data collectors and supported data entry of study measures and events, participant booking, communication between study staff and GPs, study medication tracking and upload of supporting documentation for events. *AWARD*-GP was deployed in 2011 to support recruitment staff to register associate investigators (i.e. GPs) and track recruitment activity [22]. *AWARD*-Adjudicator was deployed in 2013 to support clinical experts to complete the adjudication workflow for each clinical event. *AWARD*-AMS was deployed in 2017 to track and approve applications to access and analyse ASPREE data.

#### Technical specifications

Each *AWARD* module was a discrete web application linked to a common, secure SQL database located within an ISO 27001 certified facility at Monash University, Melbourne, Australia. All data were encrypted in transit via SSL through the ASPREE web server and Internet protocol security (IPSec) tunnels to the database cluster. Access to the *AWARD* suite was managed by the two National Coordinating Centres: Melbourne (Australia) and Minneapolis (USA). These centres were responsible for confirming the identity of staff members and ensuring staff were trained in Good Clinical Practice and the appropriate privacy protocols (e.g. Health Insurance Portability and Accountability Act of 1996). Following vetting, staff were registered as web application users by National Coordinating Centre administrators and provided with system access, which was restricted to data for their site only. Role-based access control was used to provide access to additional functionality, and roles were allocated as required. For example, the *AWARD*-Adjudicator module was visible only to staff with the endpoint adjudication role. Permission to assign user roles was limited to two system administrators. Regional supervisors were responsible for providing technical and operational training. All user activity was subject to an audit log.

### Key challenge 1 - operational support

The operational needs of ASPREE included support for traditional data entry and also support for study operations such as booking study visits for participants and communication with third parties. Maintaining a faithful digital record of the data collected on source documents at data collection visits was a key operational challenge. *AWARD* was designed to assist staff to adhere to the ASPREE protocol and standard operating procedures by providing safeguards against transcription errors and protocol deviations. Each structured data field was subject to pre-programmed value ranges, process prompts and protocol compliance checks at the point of data entry, with the aim of preventing transcription errors in real time. Staff were alerted to any out-of-range or missing values when data were saved, and prompted to double check that these data were correct. Fields relating to eligibility were subject to additional randomisation restrictions meaning that entry of data values that were outside protocol-based eligibility criteria limits resulted in inactivation of the randomisation function on the user interface. Inaccurate manual calculation of variables has been identified as a source of poor data quality [12]. Thus, wherever possible, staff entered raw data via the web application (e.g. individual blood pressure readings) and *AWARD* was programmed to calculate additional variables from the raw data (e.g. mean blood pressure).

Integrating multiple data sources to dynamically coordinate study activity is another operational support challenge. To aid the timely completion of study activity, *AWARD* also supported complex operational tasks such as visit booking. Pre-programmed venue and participant booking lists were made available to staff in real time via the web application. Participants ineligible for a visit because they were deceased, withdrawn or undergoing follow up for the dementia endpoint were automatically removed from the list when it was generated by *AWARD.* Venue booking lists included a calculation of the time required to complete outstanding activity at the venue. This calculation included a consideration of the number of participants requiring a visit, the type of visit to be conducted for each participant (e.g. a 1-h in-person visit; or a 15-min medical records search) and, if the venue was a general practice, the time required to collect supporting clinical documents. Bookings were recorded in the web application and logic checks supported staff to minimise bookings errors (e.g. participants being followed by medical records could not be booked for an in-person visit). Staff were able to communicate to general practitioners and hospitals using fax and email buttons.

### Key challenge 2 - data abstraction

Data abstraction has been shown to produce poor data quality in situations where data collectors are expected to interpret complex criteria. In ASPREE, clinical event data were manually abstracted from clinical records obtained from primary care providers, hospitals and specialists that were consequently at risk of data errors. To minimise the need for interpretation by data collectors, clinical event record forms prompted staff to transcribe key elements that comprised the event definition, rather than to interpret clinical information and record outcomes. Logic checks, particularly related to illogical dates, were implemented to screen for transcription errors. To ensure correctness, primary and secondary endpoints were adjudicated by at least two clinical experts based on raw transcribed data and PDF copies of clinical records.

### Key challenge 3 - usability

Prior to the development of the *AWARD* suite, a comprehensive understanding of a given participant’s progression in the study could only be gained by reviewing existing background data stored across a number of places. While critical in the accurate completion of certain study activities, manual integration of this information was burdensome, time-consuming and error-prone as it required traversing several web pages within the pilot data system. In response, a series of database views were programmed to retrieve data from multiple “live” SQL tables and display an appropriate value or instruction, known operationally as a “status”. Common examples of such a status include: vital status, retention status, clinical event status, dementia trigger status, participant study file status (i.e. file is with staff, in Compactus storage or archived), document request status (i.e. document requested from hospital - awaiting response) etc. These statuses were displayed prominently within the web application, which enabled viewing of all necessary information on the same page of the user interface, supporting staff to perform the appropriate action (see Additional file 1: Figure S1). Statuses also were utilised to assist in protocol compliance by displaying the operational “next steps” to be undertaken. For example, if all necessary documents had been collected for a clinical event, the event status automatically updated to an instruction to send the event for adjudication. This status was visible on relevant pages of the user interface and fed into daily reports.

Usability was supported by defining more than 20 user roles that enabled certain content within the web applications. For example, staff involved in preparing clinical events for adjudication were assigned the “Endpoint” role. This role enabled staff to view and enter data collection forms specifically related to clinical event follow up. Staff without the role could not view these forms. This ensured that staff only saw the data entry fields that they were expected to complete and were not confused by seeing fields that were not relevant to them.

### Key challenge 4 - cost-effective data querying

In line with good clinical research practice, analytical data values were subject to quality assurance processes [6]. All data included in the analysis data set were queried for missing and out-of-range values. Ranges for individual values were determined by the ASPREE International Data Management Committee (IDMC). Changes in values between visits were considered out of range if the change fell outside 3 standard deviations from the mean change in values between baseline test values and the next administration of the test (see Additional file 2: Table S1). Automated querying of data for missing or out of range values produced an “Action List” of outstanding activity for each staff member. The action list functioned as a decision support tool, alerting staff to potential data discrepancies, potential protocol deviations and any protocol-defined clinical follow up that was required, such as informing the general practitioner/primary care provider of an abnormal clinical measure (e.g. high blood pressure). These action items were prominently displayed for each user on the home page of each module of the *AWARD* suite. Actions were resolved either by updating the data entered or providing an explanatory response on the action list (e.g. data missing due to measurement device error) (see flowchart in Additional file 3: Figure S2). Responses to action list queries were monitored and if resolution of a reasonable number of queries did not identify a transcription error, query ranges were recalibrated to improve query specificity. The IDMC monitored the resolution of data queries via the action list. Data that were confirmed to be correct according to source documentation but considered to be unlikely or improbable, were reviewed and adjudicated by IDMC. Data adjudicated as implausible (outside the possible range for humans) were removed and considered to be missing due to staff error at the time of data collection.

### Longitudinal data set production

At study conclusion *AWARD-*Data and *AWARD*-Adjudicator were utilised to prepare a wide-form, longitudinal data set, following cessation of the randomised intervention on June 12 2017. The resulting data set contained more than 39 million values (*n* = 39,108,454). For transparency, the analysis data set contained the full complement of variables (i.e. for 7 years of follow up) for all participants. Some data were expected by design to be blank because the study ended or because the participant died/withdrew before data collection was scheduled, or because of the response to a parent field that precluded a response (e.g. if the participant was a non-smoker, the subsequent question about the number of cigarettes smoked was not asked and hence the data were blank). Only data that were not expected by design to be missing were included in the count of missing data. The initial primary data set for publication was locked in January 2018.

### Data quality analysis methods

The impact of *AWARD* on data quality was assessed by comparing baseline data collected from the first 1000 participants, whose data were collected using the pilot system, with the remaining participants whose baseline data were collected in *AWARD*. Descriptive statistics (numbers and percentage) were used to describe data completeness and reasons for missing data.

## Results

### Data flow in ASPREE

**Fig. 2:**
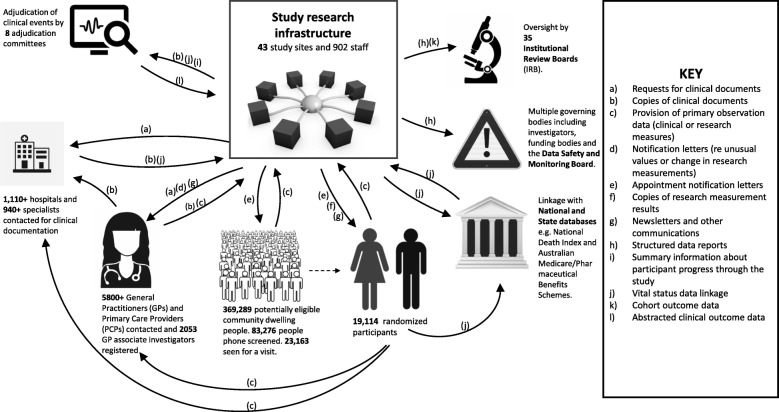
Data flow between stakeholders in the ASPREE clinical trial

*AWARD* supported data flow between numerous stakeholders from the time of study commencement in March 2010 to study closure in June 2017 (Fig. 2).

### Impact of *AWARD* on data quality

A comparison of baseline data quality between the first 1000 participants and remaining participants is presented in Table 2. The implementation of *AWARD* reduced staff errors as a cause of missing data (0.3% of entries, reduced to 0.01%), reduced the number of data values requiring query (0.14% reduced to 0.04%), and reduced the proportion of participants randomised in error (0.4% reduced to 0.08%). Overall, 65% of the first 1000 participants randomised had at least 1 (of approximately 200) baseline data values missing due to staff error. After the implementation of *AWARD,* this reduced to 2%.

**Table 2:** Data quality comparison, prior and post system upgrade to ASPREE Web Accessible Relational Database (*AWARD*)

	Prior to *AWARD*	With *AWARD*
Number of participants	1000	18,114
Number of fields collected at baseline	206	220
Baseline data missing due to staff error	646 (0.3%)	351 (0.01%)
Baseline data requiring querying	278 (0.14%)	1469 (0.04%)
Protocol deviations	4 (0.4%)	15 (0.08%)
Proportion of participants with at least 1 missing field due to staff error	65%	2%

Results are presented as number or number (percentage)

### Longitudinal data quality and completeness at scale

Currently, there is no accepted framework for the presentation of trial data quality. Table 3 details the quality of data included in the ASPREE longitudinal data set by data category. Overall, each participant contributed 1990 data values, which included all data that could theoretically be collected if all participants were followed for 7 years, along with any concomitant medications reported throughout the study. Data collection was possible for more than 15.7 million data values, including almost 1 million data values relating to eligibility. The remaining data values (*n* = 23,399,596) could not be collected because data were scheduled for collection *after* death, withdrawal of consent, or study closure; or because data were not possible as a result of the answer provided to a hierarchical question. Where data collection was possible, 96.6% of values were entered within range or found to be correct following the manual resolution of 19,787 data queries. The remaining 3.4% of data were missing. Overall, 99.9% of eligibility data were complete (i.e. baseline 3MS, Katz activities of daily living, systolic blood pressure, diastolic blood pressure and haemoglobin). Reasons for missing data are shown in Table 4.

**Table 3:** ASPREE longitudinal data quality

Data category	TOTAL potential values^ab^	Number of variables collected	Entered within range or found to be correct on querying	Unresolved queries	Protocol deviations	Values where data collection not possible^c^
			number (% of total)	number	number	
Participant demographics	824,947	80	795,984 (96.5%)	0	0	704,173
Clinical information^e^	2,413,294	227	2,358,542 (97.7%)	0	14	1,925,584
Pathology	959,347	88	842,136 (87.8%)	0	5	722,685
Medications	939,684	31^d^	937,256 (99.7%)	0	0	72,444
Family history	293,929	37	286,982 (97.6%)	0	0	413,289
Cognitive measures	2,737,448	590	2,689,837 (98.3%)	0	0	8,539,812
Physical function	509,858	73	488,648 (95.8%)	0	0	885,464
Mood, function and quality of life	6,317,845	749	6,070,867 (96.1%)	0	0	7,998,541
Endpoints	146,243	46	146,243 (100%)	0	0	733,001
Study medication	97,004	8	97,004 (100%)	0	0	55,908
Visit conduct	469,259	61	456,539 (97.3%)	0	0	696,695
TOTAL	15,708,858	1990	15,170,038 (96.6%)	0	19	23,399,596

^a^Includes all data values scheduled for collection between randomisation, death, withdrawal of consent, or study closure. Excludes fields that were not active at the time of data collection, and fields that were not applicable due to a response to a hierarchical question

^b^All values queried for missing and out-of-range data

^c^Includes all data values scheduled for collection *after* death, withdrawal on consent or study closure. Also includes fields that were not active at the time of data collection, and fields that were not applicable due to a response to a hierarchical question

^d^Plus concomitant medication. The number of medications reported varied for each participant

^e^Includes past medical history, past cancer screening, and physical examination measures such as blood pressure, heart rate, height, weight and abdominal circumference

**Table 4:** ASPREE longitudinal data completeness

Data category	TOTAL missing values^a^ number (% of total potential values)	Missing data (% of total potential values in category)				
		Visit not conducted	Third party	Participant declined	Staff/device error	Other reasons
Participant demographics	28,963 (3.5%)	1.5%	0%	2.1%	< 0.1%	0%
Clinical information	54,738 (2.3%)	1.5%	0%	0.3%	< 0.1%	0.4%
Pathology	117,206 (12.2%)	0%	10.2%	0%	0%	2.0%
Medications	2428 (0.3%)	0%	0%	0.1%	< 0.1%	0.1%
Family history	6947 (2.4%)	2.0%	0%	0.2%	0.1%	0%
Cognitive measures	47,611 (1.7%)	1.5%	0%	0.3%	< 0.1%	< 0.1%
Physical function	21,210 (4.2%)	2.6%	0%	1.5%	< 0.1%	0%
Mood, function and quality of life	246,978 (3.9%)	3.6%	0%	0.3%	0.1%	< 0.1%
Endpoints	0 (0%)	0%	0%	0%	0%	0%
Study medication	0 (0%)	0%	0%	0%	0%	0%
Visit conduct	12,720 (2.7%)	2.7%	0%	0%	0%	0%
TOTAL	538,801 (3.4%)	2.2%	0.6%	0.4%	< 0.1%	0.2%

^a^Excludes fields that were not active at the time of data collection; fields that were not applicable due to a response to a hierarchical question

Overall, failure to conduct a visit as scheduled was the most common reason for missing data (2.2% of total data were missing for this reason) and failure of a third party to provide requested data was the second most common reason. The majority of missing data values in this latter category were laboratory measures that were not performed at study visits but instead were requested from pathology testing providers and not ultimately received. Participants declining to provide information was the third most common reason for missing data. The two most commonly declined variables were demographic data (3.5% of this category were missing) and the physical function measures such as the grip strength and gait speed (4.2% of this category were missing). Staff and device error accounted for less than 0.1% of missing data overall and included data values that were considered to be implausible and removed from the database.

### Cost of *AWARD*

Development of *AWARD* is estimated to have cost US$1.1million, based on salary expenditure between 2010 and 2017 for a data manager (responsible for designing the system) and web programmers (responsible for technical development). This includes hardware and infrastructure costs. Time required to resolve an action item was calculated based on the number of queries a single staff member was able to resolve in an hour. Action item resolution took an average of 4–6 min at an estimated cost of US$8–10 per query.

## Discussion

The impact of technology on research data quality in clinical trials is poorly understood. Developed to support ASPREE, the *AWARD* suite is a custom data system that harnessed simple technology to provide innovative functionality to support data collectors to undertake complex study activities. To achieve study goals, ASPREE required a complex data flow between stakeholders that produced a small health-data ecosystem (see Fig. 2). Conduct of this large study and navigation of such a complex data flow was only made possible by the *AWARD* suite.

### Data quality and completeness

The functionality of *AWARD,* which was specifically designed to produce high-quality data, supported ASPREE to achieve 96.6% data completeness and accuracy, with the remaining data being missing. In clinical trials, some missing data is inevitable due to participant availability at the time when data is scheduled for collection or because of unavoidable participant dropout. Within the published literature, 10% missing or incorrect data is the threshold for poor data quality [6, 10, 23, 24]. ASPREE data quality is well above this acceptability threshold but further assessment of data is challenging for a number of reasons. First, data quality should ideally be compared with other similar studies, but the published literature on data quality from other, aspirin trials is limited. While most studies report the number of participants lost to follow up, the impact of this dropout on data completeness and quality is not described [25–28]. The investigators of the British Doctors Aspirin Trial state that mortality and morbidity data were considered “virtually complete” [29], but there is insufficient detail to enable comparison with ASPREE. Fowkes *et al*. describe the data management process for the Aspirin for asymptomatic atherosclerosis trial, such as double data entry into an Access database, but details of resultant data quality are not provided and hence comparative assessment is not possible [30]. Second, while data quality is universally recognised as important in health research, literature published to date has focussed on processes and practices to *produce* data quality rather than the assessment of data quality following study closure [2, 8, 12]. Thus, there is no accepted framework for reporting or assessing data quality. Given these limitations, ASPREE data quality can only be assessed in the context of the goals of the *AWARD* suite. *AWARD* was specifically designed to mitigate known contributors to poor data quality by supporting operational activity, minimising data abstraction, calculating additional variables and assisting usability. The *AWARD* suite supported ASPREE to adhere to protocol criteria (protocol deviations, *n* = 19), resolve data queries and limit missing data to well below the acceptable threshold of 10%. Consequently, we consider ASPREE data to be of high quality. Comparison of baseline data quality between the first 1000 participants whose data were collected prior to implementation of *AWARD* demonstrated that *AWARD* reduced staff error resulting in missing data, reduced the number of out-of-range data entered and reduced protocol deviations. Some of this improvement could be attributed to the increasing familiarity of the staff with the study procedures, however, this is unlikely to be the major contributing reason since staff turnover was high (~ 900 users within a 7-year period) with new staff being inducted and trained throughout the study*.* Consequently, we consider the design of *AWARD* to be instrumental in the overall data quality of ASPREE.

### Importance of operational support

Many functions of *AWARD* were designed to support operational activity such as visit bookings. Despite this, failure to complete visits as scheduled was the most common reason for missing data, accounting for 65% of all the missing data. While *AWARD* design features for data entry, data abstraction and staff decision support were informed by published literature, operational support functionality in *AWARD* was designed in house without peer-reviewed guidance. Further research and innovation regarding successful methods for supporting study operations may improve operational performance and limit missing in future trials.

### Cost effectiveness

Despite improvements associated with electronic data collection [13], data cleaning is still considered to be an expensive process. It is generally anticipated that clinical trials generate 2–3 data queries per electronic data capture form and that each query costs US$100 to resolve [6]. According to these figures, ASPREE would have been expected to produce between 2.6 and 4 million data queries, at a massive cost to the study. Due to the checks and balances included in *AWARD* by design, only 19,787 values required manual checking against the source documentation, over the course of the entire study (0.1% of all potential values). Fine tuning of the range for change over time queries could have further reduced this number. This was considerably fewer than expected, significantly reducing the cost of data cleaning. Implementation of the action list system allowed staff to resolve data queries within 4–6 min or $US8–10, a cost saving reduction compared with the standard US$100 cost per query. This was only possible because of the underlying data entry, operational infrastructure and functionality of *AWARD*, which took time and significant funds to develop. While the development cost of *AWARD* was low in comparison to the cost of the study as a whole (~ 2% of grant award), the authors recognise that development of a custom $1.1 million system is beyond the scope of many clinical trials and more technology solutions are now available to clinical trialists, both commercial and freeware. However, commercial options can also be prohibitively expensive and geared towards billing support more so than operational activity support. Freeware options are capable of meeting a fair portion of study needs but often lack comprehensive functionality. Thus, trialists relying on freeware options must link multiple products in order to construct a more complete solution, which has its own drawbacks. Furthermore, in a recent review of clinical trial technology, none of 19 systems examined completely supported the data management needs of clinical trials [31]. We suggest that the *AWARD* suite provides proof of principle that user-centred design can produce high-quality data by supporting operational activities. Future clinical trials using commercial or freeware clinical trial data systems should also consider whether similar data collection support features to those described here can be implemented or developed within their systems.

### Strengths, limitations and novelty of *AWARD*

#### Strengths

Key strengths of *AWARD* included user-driven system design, detailed data quality annotation and custom design. Together, these features enabled *AWARD* to support the entire lifecycle of the ASPREE project by providing operational support functionality in additional to carefully controlled data entry.

#### Limitations

As a custom system, *AWARD* has limitations. Development of *AWARD* required the engagement of web programmers to hard-code functionality. At times this created a *bottleneck* that meant that rapid implementation of new functionality was not possible. A system designer with expertise in both medicine and technology was required to consult with data collectors and design usable functionality. Availability of this expertise was a limitation. Additionally, while informal feedback on usability was sought from staff, we did not collect formal technology usability assessment data.

#### Novelty

The fact that *AWARD* successfully serviced the operational complexities of ASPREE is in itself novel given that many other systems cannot provide for the complete needs of a clinical trial [31]. Achieving this functionality was made possible by the novel design methodology that focussed on supporting data collectors. The detailed data quality reporting capability of *AWARD* enabled demonstration that this design framework did indeed produce higher-quality data.

## Conclusion

The *AWARD* suite is a system that was custom built to meet the needs of ASPREE’s data ecosystem. *AWARD* provides proof of principle that designing technology to support data collectors can mitigate known causes of poor data quality; produce higher-quality data and facilitate highly detailed reporting. Pre-data collection activities (e.g. visit booking) are a key area for improvement in the technical support of clinical trials. Health IT products whether they be commercial, freeware or custom (such as *AWARD)* supporting the conduct of operational activity in addition to traditional data entry will be of enhanced use to community-based clinical trials. A standardised framework for reporting data quality would aid comparison of data quality across trials.

## Supplementary information

**Additional file 1: Figure S1.** *AWARD*-Data system screen capture showing display of key operational statuses.

**Additional file 2: Table S1.** Reference ranges and query logic for out-of-range data queries performed on numeric data.

**Additional file 3: Figure S2.** Flowchart of data query resolution process using the *AWARD* Action List.

## Data Availability

The datasets used and/or analysed for this publication are available via the ASPREE Principal Investigators. Requests for data access can be directed to aspree.ams@monash.edu.

## Abbreviations

3MS: Modified Mini Mental State Examination
ASPREE: Aspirin in reducing events in the elderly
*AWARD*: ASPREE Web Accessible Relational Database
*AWARD*-Adjudicator: ASPREE Web Accessible Relational Database - Adjudicator module
*AWARD*-AMS: ASPREE Web Accessible Relational Database - Access Management System module
*AWARD*-Data: ASPREE Web Accessible Relational Database - Data module
*AWARD*-GP: ASPREE Web Accessible Relational Database - General Practice module
EHR: Electronic health record
GP/PCP: General practitioner or primary care provider
IDMC: International Data Management Committee
