# Supplementary figures and images for: Overcoming challenges to data quality in the ASPREE clinical trial
Source: Trials. 2019 Dec 9;20:686. doi: 10.1186/s13063-019-3789-2 (PMC6902598; doi:10.1186/s13063-019-3789-2)

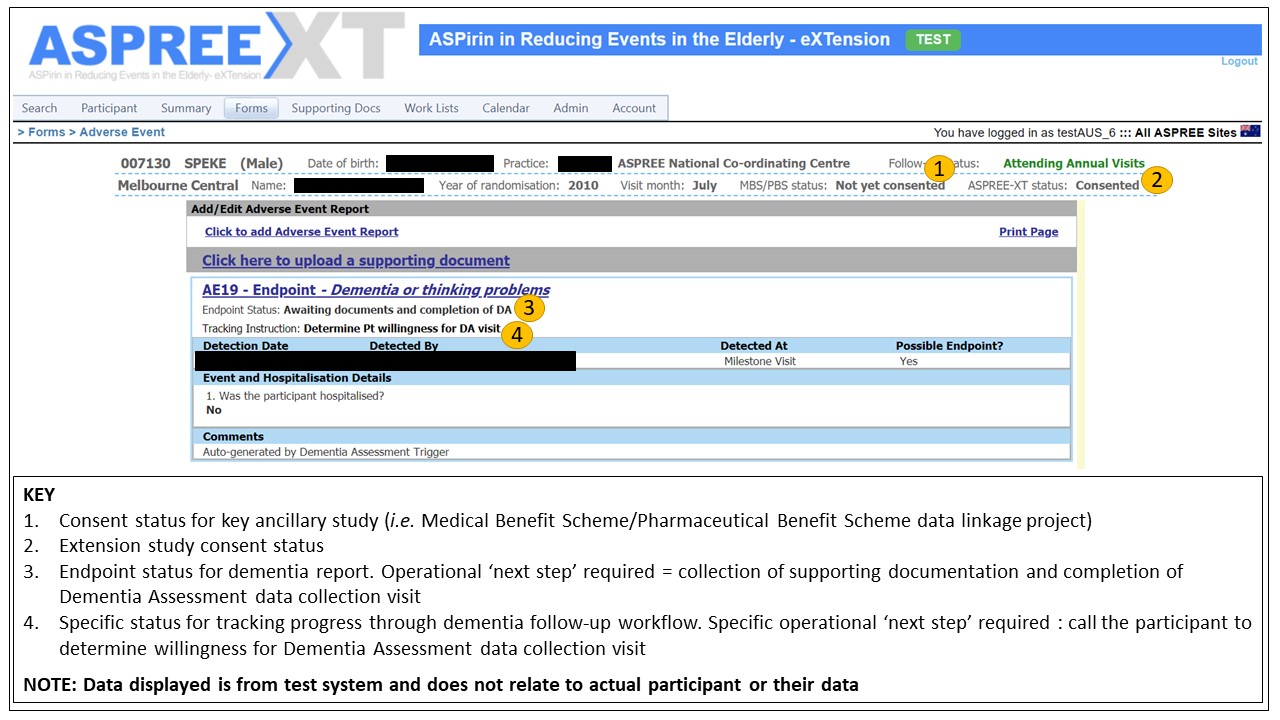

Supplement: Supplementary file 1 — Additional file 1: Figure S1. AWARD-Data system screen capture showing display of key operational statuses. [file 13063_2019_3789_MOESM1_ESM.jpg]
