# Supplementary material for: Overcoming challenges to data quality in the ASPREE clinical trial
Source: Trials. 2019 Dec 9;20:686. doi: 10.1186/s13063-019-3789-2 (PMC6902598; doi:10.1186/s13063-019-3789-2)
Supplement: Supplementary file 2 — Additional file 2: Table S1. Reference ranges and query logic for out-of-range data queries performed on numeric data. [file 13063_2019_3789_MOESM2_ESM.pdf]

Table S1: References ranges and query logic for out-of-range data queries performed on numeric data

Field#	Value range*	Change over time query range^
All Data	Missing values	N/A
All Data	Illogical values	N/A
US Total Cholesterol	70 - 375 mg/dL	3 standard deviations from the mean change between baseline and year 1
US Low-density Lipo-protein	20 - 375 mg/dL	
US High-density Lipo-protein	0.1 - 300 mg/dL	
US Triglycerides	0.1 - 850 mg/dL	
US Creatinine	0.5 - 1.7 mg/dL	
US Glucose	35 - 250 mg/dL	
US Haemoglobin	12 - 25 g/dL	
US Urine Albumin Creatinine ratio	0.8 - 665 mg/g	
AUS Total Cholesterol	2.0 - 10 mmol/L	
AUS Low-density Lipoprotein	0.5 - 10 mmol/L	
AUS High-density Lipoprotein	0.1 - 8 mmol/L	
AUS Triglycerides	0.1 - 9.9 mmol/L	
AUS Creatinine	40 - 150 µmol/L	
AUS Glucose	2 - 15 mmol/L	
AUS Haemoblogin	Female 11 - 25 g/dL<br>Male 12 - 25 g/dL	
AUS Urine Albumin Creatinine Ratio	0.1 - 75 mg/mmol	
Controlled Oral Word Assocation Texst score	1 - 40 score	
Symbol Digit Modalities Test score	1 - 100 score	
Grip strength 1st hand	5 - 65	
Grip strength 2nd hand	5 - 65	
Gait speed test 1	1.6 - 15 seconds	
Gait speed test 2	1.6 - 15 seconds	
Weight	35 - 140 kg	
Abdominal circumference	45 - 145 cm	
Height	1.4 - 2.1 m	3 standard deviations from the mean change between baseline and year 5 (when height was re-measured)
Systolic blood pressure - first measure	90 - 200 mmHg	3 standard deviations from the mean change between the average SBP at baseline and the average SBP at year 1
Systolic blood pressure - second measure	90 - 200 mmHg	
Systolic blood pressure - third measure	90 - 200 mmHg	
Diastolic blood pressure - first measure	50 - 110 mmHg	3 standard deviations from the mean change between the average DBP at baseline and the average DBP at year 1
Diastolic blood pressure - second measure	50 - 110 mmHg	
Diastolic blood pressure - third measure	50 - 110 mmHg	
Hearrt rate - first measure	40 - 120 bpm	3 standard deviations from the mean change between the average HR at baseline and the average HR at year 1
Heart rate - second measure	40 - 120 bpm	
Heart rate - third measure	40 - 120 bpm	

The above Table shows the variables that were subject to out-of-range value and out-of-range change over time queries at every point of data collection (i.e. at baseline and all data collection visits thereafter).

# Variable within AWARD that was queried for out-of-range values and out-of-range change over time.

** Acceptable range. Values below or above the cutoffs provided were considered to be out-of-range and were specifically checked against source documentation.*

^ Logic for determination of data that were considered out-of-range based on previously entered values. These data (both the data in question and the previously entered data) were checked against the source documentation.
